# Management with Wrap Disruption after Nissen Fundoplication in a Child with Gastro-oesophageal Reflux After Congenital Oesophageal Atresia: A Case Report and Minireview

**DOI:** 10.34763/jmotherandchild.20202404.d-20-00013

**Published:** 2021-07-16

**Authors:** Aleksandra Dybowska, Paulina Kupniewska, Renata Kuczyńska, Magdalena Tworkiewicz, Przemysław Gałązka, Anna Szaflarska-Popławska, Aneta Krogulska

**Affiliations:** 1 SRC Paediatric Allergology and Gastroenterology, Department of Paediatrics, Allergology and Gastroenterology, Ludwik Rydygier Collegium Medicum in Bydgoszcz, Nicolaus Copernicus University Torun, Torun Poland; 2 Department of Paediatrics, Allergology and Gastroenterology, Ludwik Rydygier Collegium Medicum in Bydgoszcz, Nicolaus Copernicus University Torun, Torun Poland; 3 Department of General and Oncological Surgery for Children and Adolescents, Ludwik Rydygier Collegium Medicum, Bydgoszcz, Nicolaus Copernicus University in Torun, Torun Poland; 4 Department of Paediatric Endoscopy and Gastrointestinal Function Testing, Ludwik Rydygier Collegium Medicum in Bydgoszcz, Nicolaus Copernicus University in Torun Torun Poland

**Keywords:** atresia, children, gastro-oesophageal reflux, Nissen fundoplication, oesophageal

## Abstract

Pathological gastro-oesophageal reflux (GER) is one of the most common complications that results in the aftermath of treatment of congenital oesophageal atresia (EA). The aim of this study is to present a case of a 7-year-old girl with severe gastro-oesophageal reflux disease (GERD) operated on in the neonatal period due to EA with a lower tracheo-oesophageal fistula (TEF). The patient, despite the use of adequate conservative treatment, clinically and in the endoscopic examination was diagnosed with severe oesophagitis (LA-D in the Los Angeles classification). After a laparoscopic fundoplication by the Nissen method at the age of 4, a transient clinical improvement and a reduction of inflammatory lesions in the oesophagus were obtained. Three years after the procedure, the patient presented with deterioration of GERD clinical symptoms in the form of: regularly occurring vomiting with periodic admixture of fresh blood, recurrent cough, symptoms of dysphagia and failure to thrive. An upper gastrointestinal endoscopy (upper GI endoscopy) revealed significant progression of inflammatory changes in the oesophagus and the two-level oesophageal stricture together with endoscopic signs of wrap disruption. Based on the conducted diagnostics, the girl was qualified for surgical revision. The diagnosis was confirmed intraoperatively. During the 4-month postoperative period, a significant clinical improvement and resolution of symptoms were observed. The presented case indicates the need for close and long-term monitoring of patients after EA. In the case of a recurrent reflux oesophagitis in patients after anti-reflux surgery, the possibility of prolonged complications, such as a wrap disruption, herniation or slippage should be taken into consideration.

## Introduction

Oesophageal atresia (EA) is a birth defect of the oesophagus that consists of its lack of continuity. It occurs in the European population with a frequency of 1–2 out of 5000 live births.^1^ This defect is most often accompanied by a lower tracheooesophageal fistula (TEF), in about 86% of cases.^2^ Although corrective surgery restoring oesophageal continuity is carried out in the first days of a child’s life, patients often suffer life-long complications associated with a congenital anomaly or possibly resulting from a surgical treatment.^1^, ^3^, ^4^, ^5^ Gastro-oesophageal reflux (GER) is the most common late complication, which affects 33–58% of patients with a history of a congenital EA.^6^ Clinical manifestation of GER can show various symptoms – from vomiting, regurgitation, heartburn, swallowing disorders or coughing, failure to thrive, recurrent respiratory tract infections and anaemia.^1^, ^5^, ^7^, ^8^ GER may have a chronic course, requiring long-term pharmacological treatment.^3^ According to the recommendations of the European and North American gastroenterological societies (ESPGHAN-NASPGHAN), all patients after EA should be treated with proton pump inhibitors (PPIs) until the first year of life, which should be continued in the event of persistent symptoms of GER. GERD complications can occur not only in childhood but also in adolescence and adulthood, and even in previously asymptomatic patients. This requires regular endoscopic examinations in all patients after EA.^8^

In the pathogenesis of GER, in patients after treatment of EA, an important role is played by the physiological anti-reflux barrier disorder resulting not only from the presence of the defect itself but also from the specifics of the repair surgery. Surgical reconstruction of the gastrointestinal tract causes shortening of the oesophagus, which may result in the displacement of the gastro-oesophageal junction upwards, and consequently a change in the angle of His.^7^, ^9^ The occurrence of GER is additionally favoured by oesophageal motility disorders coexisting with the defect, lack of peristalsis at the anastomosis and hiatal hernia.^6^, ^7^ Conservative treatment of GERD in patients operated on for EA may be ineffective and 10–45% of patients require surgery – Nissen fundoplication.^9^ Anatomical abnormalities underlying GER cause that the effectiveness of the anti-reflux surgery is lower in patients after EA, and more often those patients require re-fundoplication compared to patients without EA– 18% and 7%, respectively.^7^ The aim of this study is to present the case of a 7-year-old girl with severe GERD after a correction of the congenital EA (type C with lower TEF) in the neonatal period.

## Case report

A 7-year-old girl, L.S., operated on in the neonatal period due to EA, was referred to the clinic because of daily vomiting with an admixture of fresh blood for a month.

In her perinatal history she was born on time, by vaginal delivery, aprenatally polyhydramnios was noted. The birth weight was 2590 g. The child was scored on the Apgar scale with 7/8/9 points in the 1, 3 and 5 min, respectively. In the first day of life, the girl had feeding difficulties, drooling and progressive respiratory failure. A chest x-ray was performed with the catheter inserted into the oesophagus and filled with a water soluble contrast medium. EA with the lower tracheooesophageal fistula was diagnosed. On the second day of life, a surgical correction of EA TEF was carried out. Intraoperatively a typical (type C) EA with lower TEF was confirmed. The gap length between the upper and lower oesophageal segments was approximately 1–1.5 cm. Using the right posterior thoracotomy (extrapleural approach), fistula was divided and closed with stitches and oesophagus anastomosed end-to-end with minimal tension. According to surgical protocol, at seven postoperative days a feeding through the gastric tube was introduced and after two weeks, an oesophageal X-ray contrast study was performed and feeding per OS was started. There were no surgical complications regarding early postoperative period. Oesophageal contrast study was carried out additionally for three times in her infancy with two oesophageal calibrations. According to surgical and radiological opinion there was a need for repetitive dilatations. Due to history of EA and the view of open Hiss angle on oesophageal study it was recommended to take omeprazole (1 mg/kg bw/24 h). The girl was transferred and kept under gastroenterological care in the outpatient mode. In the first 2 years of life, the girl vomited periodically.

During an upper GI endoscopy performed at the age of 2, gastro-oesophageal reflux oesophagitis C in Los Angeles classification (LA-C) was diagnosed, and additionally the tortuous oesophagus course was described. It was recommended to continue the treatment with omeprazole (1 mg/kg bw/24 h). Vomiting continued periodically.

After a year of treatment with omeprazole, at the age of 3, a control upper GI endoscopy was performed, which revealed a high grade oesophagitis (LA-D) and also noted the lack of pronounced oesophageal peristalsis. The oesophageal lumen did not show stenoses. Based on histopathological findings, eosinophilic and infectious oesophagitis were excluded. In addition, an open cardia and features of an oesophageal hiatal hernia were found.

In the absence of the effectiveness of the conservative pharmacological treatment, the patient was qualified for a surgery, which was carried out at the age of 4. An intraoperative report confirmed severe inflammatory changes in the region of a gastric cardia. Laparoscopic Nissen fundoplication was performed according to the standard surgical technique. Oesophageal hiatus of the diaphragm was reconstructed and a fundoplication wrap created using non-absorbable braided sutures. There were early postoperative complications. It was recommended to take omeprazole (1 mg/kg bw/24 h) for a month. After surgery, the frequency of vomiting decreased, but it did not completely disappear. Owing to the need of close monitoring of the patient, a control upper GI endoscopy was scheduled. X-ray study was abandoned on the grounds of radiological protection. A follow up of upper GI endoscopy performed 4 months after the procedure described persistent high-grade oesophagitis (LA-D), and additionally showed two oesophageal constrictions freely passing a 6 mm endoscope – one at the site of the anastomosis and the other in the lower oesophagus of a possible inflammatory etiology. No flaccid gastric cardia was noted. Omeprazole (1 mg/kg/24 h) was recommended again, with a clinical improvement in the form of a reduced vomiting.

For the next 3 years, the patient was under the outpatient care of a paediatric surgeon and a paediatric gastroenterologist. During this time she was treated with omeprazole (1 mg/kg bw/24 h). During follow-up visits, she reported chest pain, neck pain, the need to interrupt a meal, cough and vomiting. Symptoms occurred periodically and were of a varying severity, which included postprandial vomiting that was the most troublesome. A physical examination revealed an impairment of a nutritional status (BMI 12.3 kg/m^2^; ?3c). An upper GI endoscopy (2 years after the fundoplication) did not described active inflammatory changes in the oesophagus; only polypoid postinflammatory lesions were visible, the cardia was tight and stenoses were not described. Omeprazole was discontinued when no worsening of the symptoms were observed. At the age of 6, in another clinic, the child was diagnosed with asthma and inhaled budesonide was recommended. An upper GI endoscopy (3 years after the fundoplication) described inflammation of the mucosa in the lower oesophagus (LA-C), cardia flaccidity and stenosis at the anastomosis site. Omeprazole (1.3 mg/kg/24 h) and additionally trimebutin (15 ml/24 h) were recommended as therapy. For half a year of conservative treatment, the clinical symptoms remained at the same level.

**Figure 1:**
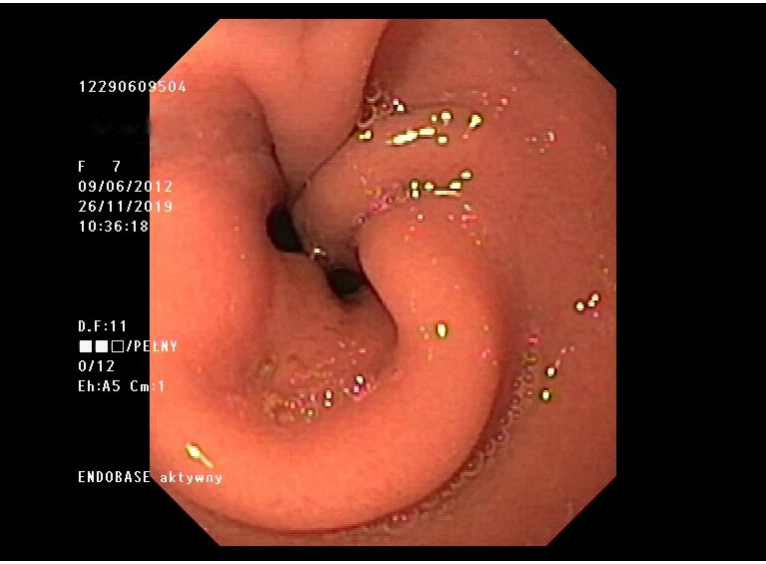
Endoscopic view with suspicion of wrap disruption. The cardia was flaccid and remained open during the examination.

**Figure 2:**
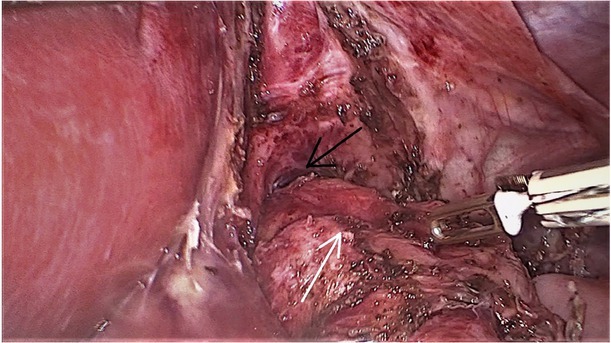
Initial intraoperative view. Severe adhesions in the area of abdominal oesophagus were released. Black arrow indicates crural disruption, while white arrow indicates disrupted Nissen wrap.

**Figure 3:**
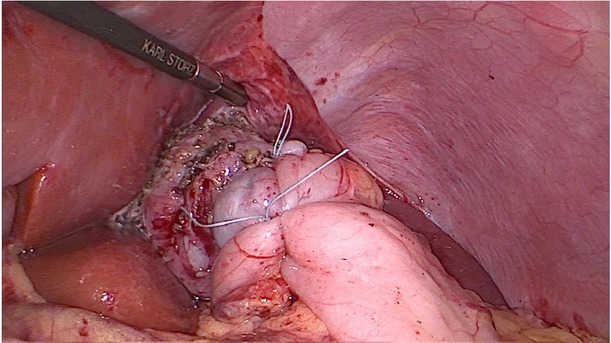
Final intraoperative view. Redo Nissen fundoplication was done.

A month before the current hospitalisation, the symptoms increased. Vomiting occurred every day, most often during or after a meal, and fresh blood was seen in the emetic content. The child also reported pain in the umbilical region of the abdomen, a feeling of regurgitation, a feeling of obstruction in the oesophagus, coughing and choking during a meal, an unpleasant smell from the mouth. Despite good appetite, BMI decreased to 12 kg/m^2^. Additional tests revealed microcytic anaemia. An upper GI endoscopy (performed 8 months from the previous one) found a grade D severe oesophagitis (LAD), two oesophageal stenoses – the first at a height of 15 cm from the incisor teeth, which corresponded to a surgical anastomosis, while the second at a height of 23 cm from the incisor teeth – inflammatory. The cardia was flaccid, and of a twisted shape. It did not surround the endoscope closely and remained open during the examination (Figure 1). Owing to a significant progression of lesions in the oesophagus, the patient was qualified for laparoscopic re-fundoplication. Intraoperatively, the disruption of the fundoplication cuff in the lower part of the cuff and loosening of the seams in its upper part were confirmed (Figures 2 and 3). After the procedure, the patient tolerated a mechanically soft diet well and did not report any symptoms. As part of the postoperative treatment, omeprazole (1.3 mg/kg/24 h) was recommended. A follow-up of the upper GI endoscopy was scheduled in six months.

## Discussion

Patients operated on due to EA should be followed up by a multidisciplinary team. Author`s centre is a regional reference centre for newborn surgery as well as paediatric gastroenterology. Since 2013, all newborns with EA TEF have been operated on thoracoscopically. The described patient was one of the last operated through toracotomy. Despite the repair surgery, they may have chronic gastrointestinal and respiratory symptoms.^4^, ^5^, ^8^, ^10^ The most common respiratory symptoms are coughing, wheezing and shortness of breath. In addition, patients may suffer from recurrent respiratory infections.^3^, ^5^ Respiratory symptoms, especially if they appear isolated, always require an exclusion of the anatomical abnormalities, such as laryngeal cleft, tracheomalacia, vascular ring or vocal cord paralysis.^8^ Our patient’s clinical concerns (coughing and bronchial obstruction) did not indicate the above abnormalities. However, the child was diagnosed with asthma. Of the gastrointestinal complaints, the most common are: dysphagia and GERD symptoms.^5^

The abnormal anatomical conditions and the history of gastrointestinal tract restoration in patients operated on in the neonatal period due to EA cause a disorder of the natural anti-reflux barrier.^7^, ^9^ Therefore, the effectiveness of GERD conservative treatment in patients after EA is limited as we observed in our patient.

Patients with persisting clinical symptoms, despite proper conservative therapy, patients with recurrence of aspiration symptoms and nutrition disorders and patients with severe oesophagitis after an upper GI endoscopy are recommended for anti-reflex surgery.^7^, ^8^, ^11^ Especially, patients with a long-gap EA and recurrent stenosis at the anastomosis site can benefit from such treatment.^8^ The presented patient therefore met the eligibility criteria for the surgical treatment and was a good candidate to achieve benefits from the surgery.

Nissen fundoplication in most patients initially improves the clinical condition, but in some operated patients this effect is unstable, which may be due to the disruption of the fundoplication cuff. In these cases, two strategies are to be considered – a conservative treatment or a subsequent surgery. Long-term conservative therapy with a PPI allows in many patients to control the symptoms of oesophagitis and achieve endoscopic remission.^9^ It should be taken into account that chronic inhibition of a gastric acid secretion may increase the risk of upper respiratory tract infection and pneumonia.^8^ In addition, hypochlorhydria causes intestinal dysbiosis, which predisposes to intestinal infections.^12^ In turn, re-fundoplication, although technically more difficult than a primary surgery, is successful in 70–80% of patients.^9^ Author’s centre has a vast experience experience in gastro-oesophageal reflux surgery with 28 cases were performed laparoscopically or through laparotomy in the last 4 years. The main group of those patients include neurologically impaired children followed by children after EA. Our described patient was the second one requiring revision after laparoscopic fundoplication, and the other one was patient with history of severe prematurity and post haemorrhagic hydrocephalus. The hydrocephalus patient also presented with Nissen wrap dislocation after severe vomiting in the course of ventriculo-peritoneal shunt disfunction.

Our patient’s fundoplication performed at 4 years of age brought about a clinical improvement. An upper GI endoscopy performed 4 months after the surgery confirmed that the endoscope was tightly cuffed with the fundoplication, but severe oesophagitis was still present, and therefore PPI was reintroduced. Thanks to a combined treatment, (i.e. surgical and pharmacological), clinical and endoscopic remission was achieved in subsequent years. In an upper GI endoscopy 2 years after the fundoplication, no active inflammatory changes or cardia insufficiency were observed, which was a reason for a discontinuation of the omeprazole treatment. In some patients, during follow up, the fundoplication cuff may loosen and the associated recurrence of clinical and/or endoscopic symptoms may be seen.^6^, ^7^ This complication occurred in our patient 3 years after the first surgery. The child demonstrated not only periodic vomiting but also a cough that could promote the dissolution of the fundoplication cuff. It cannot be excluded that microaspiration has occurred. In addition to the progression of inflammatory lesions in the oesophagus, flaccid cardia was reported in an upper GI endoscopy. However, an attempt was made with a conservative treatment, also because the parents did not accept the initial proposal of surgery. Finally, after several months, due to the deterioration of clinical (bloody vomiting) and endoscopic (severe oesophagitis) symptoms, the child was qualified for the reoperation. Re-fundoplication was performed, resulting in the resolution of all previously reported symptoms. Further observation will assess the long-term effectiveness of the treatment used.

Gastrointestinal and respiratory complications in patients with EA (aspiration episodes, oesophageal motility disorders, GER, oesophagitis and narrowing of surgical anastomoses, respiratory failure) trigger feeding difficulties. The problem may be a fear of eating because of repeated choking episodes and swallowing disorders. The above conditions could have been the cause of our patient’s nutritional deficit. ESPGHAN-NASPGHAN recommendations indicate the lack of specific methods to prevent feeding difficulties in patients with EA. Attention was drawn to the need for a multi-specialist management in these cases and to the need of an early inclusion of enteral and oral nutrition in newborns after EA surgery as interventions to reduce the risk of malnutrition later in life.^8^

When presenting the case of our patient, we would like to draw attention to the complexity and chronicity of health problems occurring in a child operated on in the neonatal period due to EA. The correlation of clinical symptoms with endoscopic changes is not always complete. Initially, our patient experienced only periodic vomiting, although severe oesophagitis was found. However, an upper GI endoscopy performed 2 years after the first surgery did not reveal any active inflammatory changes in the oesophagus despite the intensified clinical symptoms. This indicates the need for a regular endoscopic supervision already at an early stage of follow up after EA, despite the additional burden of both repeated upper GI endoscopies and a general anaesthesia. It should also be emphasised that the treatment should be optimised depending on the macroscopic changes in the oesophagus, not just the clinical symptoms. EA is a defect that is long-term associated with potential complications – in our case, severe reflux oesophagitis. Persistent significant oesophagitis despite a long-term conservative treatment and ultimately requiring double surgery in the early years of a child’s life, creates a risk of further distant complications such as Barrett’s oesophagus and oesophageal cancer.^5^, ^7^, ^8^, ^13^ The risk of oesophageal adenocarcinoma is 50 times higher in patients with EA compared to the population risk, and the diagnosis concerns people at a relatively young age.^4^, ^7^ Information on late complications of EA and GERD should be given to both the child’s parents and the family doctor. Gastroenterological recommendations recommend routine endoscopic monitoring (every 5–10 years) in adults with a history of EA, even in the absence of symptoms.^8^ A close cooperation with parents is advised to obtain a good adherence to the recommendations.

## Conclusions

The described case indicates the need for a close and longterm monitoring of patients after the EA surgery. In the case of relapses of reflux oesophagitis in patients after anti-reflux surgery, the possibility of distant complications such as dislocation or dissolution of the fundoplication cuff should be taken into consideration.

## Author contributions

All authors were substantially involved in drafting the work, revising it critically for important intellectual content; final approval of the version to be published; agreement to be accountable for all aspects of the work in ensuring that questions related to the accuracy or integrity of any part of the work are appropriately investigated and resolved. RK, MT and PG were involved in acquisition and interpretation of data for the work. AD and PK were involved in analysis, conception and design of the work. AK and AS-P revised the manuscript and had substantial contributions to the conception.
